# Academic Publication of Neurodegenerative Diseases From a Bibliographic Perspective: A Comparative Scientometric Analysis

**DOI:** 10.3389/fnagi.2021.722944

**Published:** 2021-11-04

**Authors:** Zhenxin Liao, Wei Wei, Mengling Yang, Xuyuan Kuang, Jian Shi

**Affiliations:** ^1^Department of Pediatrics, The Third Xiangya Hospital, Central South University, Changsha, China; ^2^School of Public Health, Central South University, Changsha, China; ^3^Department of Radiology, The Third Xiangya Hospital, Central South University, Changsha, China; ^4^Department of Hyperbaric Oxygen, Xiangya Hospital, Central South University, Changsha, China; ^5^Department of Spine Surgery, The Third Xiangya Hospital, Central South University, Changsha, China

**Keywords:** neurodegenerative diseases, aging neuroscience, bibliometric analysis, comparative scientometric analysis, visualization, women in neuroscience

## Abstract

**Background:** For measuring the impact in clinical and scientific research, the citation count of the articles is used in the bibliometric analysis, although there is no comprehensive summary of neurodegenerative disease research. This study intends to provide the neuroscientists and investigators with a practical reference guide to appraise the most important and influential articles written on this subject through a macroscopic view of the research activities on neurodegenerative diseases.

**Materials and Methods:** The Clarivate Analytics Web of Science was searched in July 2020. To ensure the breadth of the search scope, the search terms were confirmed as “multiple sclerosis” (MS) or “amyotrophic lateral sclerosis” (ALS) or “Parkinson's” or “Alzheimer's” or “Huntington's” or “neurodegenerative.” After excluding completely unrelated articles, the top-cited articles were collected and evaluated from special characteristics. The data analysis was performed using SPSS 18.0. The articles were characterized by citation number, publication year, topic, study type, authorship, journal, country, and institute of responding author and foundation.

**Results:** The query identified 593,050 articles. A total of 45% of the top-cited articles were published during 2000–2009, followed by 30 articles from 1990–1999. Diagnosis and pathology were the main research categories (*n* = 62). Alzheimer's disease (AD) was the main study topic (*n* = 43). Meanwhile, the United States confirmed the tremendous impact on the field of neurodegenerative diseases. Notably, 69 of 100 articles were studied in the United States, and the National Institutes of Health sponsored 49 articles. There were only 22 articles that can be divided by evidence level. No article was categorized as level 1 evidence. In the journal list with multiple articles, seven of 15 were general journals. The 58 authors, who contributed to more than one article, have been identified by VOSviewer, and the clusters of authors reveal the evolution of research focus in neurodegenerative diseases.

**Conclusions:** This study analyzed the bibliometric characteristics and connections of 100 top-cited articles in the field of neurodegenerative diseases in the Web of Science. Their main outcomes were as follows: First, the pathology and diagnostic researches took a major role in top-cited articles while the therapy articles are relatively less. Second, the United States confirmed the tremendous impact on the field of neurodegenerative diseases. Third, researchers also submitted their researches to general journals, not just focused on specialty journals.

## Introduction

Neurodegenerative diseases, which are characterized by the progressive loss of neurons in the central nervous system, affected more than 50 million people worldwide (Nuic et al., 2018; Ehrenberg et al., 2020). At present, no treatment exists to arrest or slow down the neurodegenerative process (Du et al., 2015). Moreover, due to global demographic changes and life expectancy increases in humans (Kepp, 2019), aging-related neurodegenerative diseases are ever-increasing (Agarwal et al., 2016). Bibliometric sciences offer both a statistical and quantitative analyses of published articles and provide a measurement of their impact in a particular field of research (Baier-Fuentes et al., 2020). This field of science has continuously evolved since 1987 (Garfield, 1987; Ahmad et al., 2014), but the bibliometric analysis in neurodegenerative diseases has not been reported.

As a quantitative study of previously published articles, the bibliometric analysis is used to study various aspects of science (Ellegaard and Wallin, 2015; Du et al., 2020). An analysis of the top 50 or 100 most-cited articles can help researchers review the research history in a specific field and make further contributions based on the classic literature. Thus, we chose the 100 top-cited articles to propose a representative list of intellectual milestones in the field (Du et al., 2020). A few of the top-cited studies in the field of the neurodegenerative disease have been reported. In 2009, a bibliometric analysis related to Alzheimer's disease (AD) has been reported by Sorensen. In 2018, Xue et al. reported “The 100 most-cited articles in Parkinson's disease” (PD) (Xue et al., 2018) for the first time. However, no top-cited bibliometric analysis has been conducted on the whole field of the neurodegenerative disease.

Since the middle of the twentieth century, specialists and researchers are committed to provide hypotheses into neurodegenerative diseases. An increasing number of articles are published annually to show *in vitro* experiments, animal experiments, and clinical applications in this field. Given that, this study aimed at determining a ranking of the 100 top-cited articles regarding all kinds of neurodegenerative diseases. This study can help researchers better determine the direction of their study, meanwhile further analyze for better understanding of the qualities of classical studies and special characteristics of highly cited articles, and highlight the significant contribution of these studies to the field of neurodegenerative diseases.

## Materials and Methods

### Confirmation of Database and Search Scope

To recognize the 100 top-cited articles related to the field of neurodegenerative diseases, the authoritative and professional citation indexing database, i.e., Web of Science, was used on July 22, 2020 (Web of Knowledge, 2020). To ensure the breadth of the search scope, the search terms were confirmed as “multiple sclerosis” (MS) or “amyotrophic lateral sclerosis” (ALS) or “Parkinson's” or “Alzheimer's” or “Huntington's” or “Neurodegenerative.” The document type has no restrictions, and the language has been restricted to English only.

### Recognition of the 100 Top-Cited Articles

After confirming the search scope, 593,050 articles were expressed in descending numerical order according to the citations. The top 150 articles have been exported, the full text has been read in order by two researchers, respectively. After excluding six articles that (1) were completely irrelevant to the neurodegenerative disease and (2) mentioned the neurodegenerative diseases while focusing on irrelevant topics, the 100 top-cited articles were confirmed (Supplementary Table 1).

### Extraction and Analysis of the Data

The full record contents were exported from the Web of Science in the format of both plain text (for the analysis in VOSviewer) and Tab-delimited (for the manual check and analysis). The basic information included citation number, publication year, topic, study type, authorship, journal, country, and institute of responding author and foundation. The VOSviewer is a software for creating maps such as coauthorship, co-occurrence, citation, bibliographic coupling, or co-citation links. Other than mapping, all the contents have been checked manually, and the descriptive analysis has been performed using SPSS Statistics for Windows (Armonk, NY: IBM Corp.). The authors of each article were extracted, and the connection between authors was analyzed using VOSviewer (developed by Nees Jan van Eck and Ludo Waltman at Leiden University's Centre for Science and Technology Studies, Leiden, Holland) (version 1.6.15). Items have been grouped into clusters according to the total link strength attribute, which indicates the total strength of the co-authorship links of a given researcher with other researchers. Also, the color of the item is determined by the publication year of their articles. The algorithms of VOSviewer have been explained in the previous study (Van Eck and Waltman, 2017). The evidence levels of the top-100 articles have been determined according to the rating criteria (Guyatt et al., 2011). We read the full text of each article and tried to match each article from level 1 to level 5 according to the description of the rating criteria.

## Results

**Figure 1:**
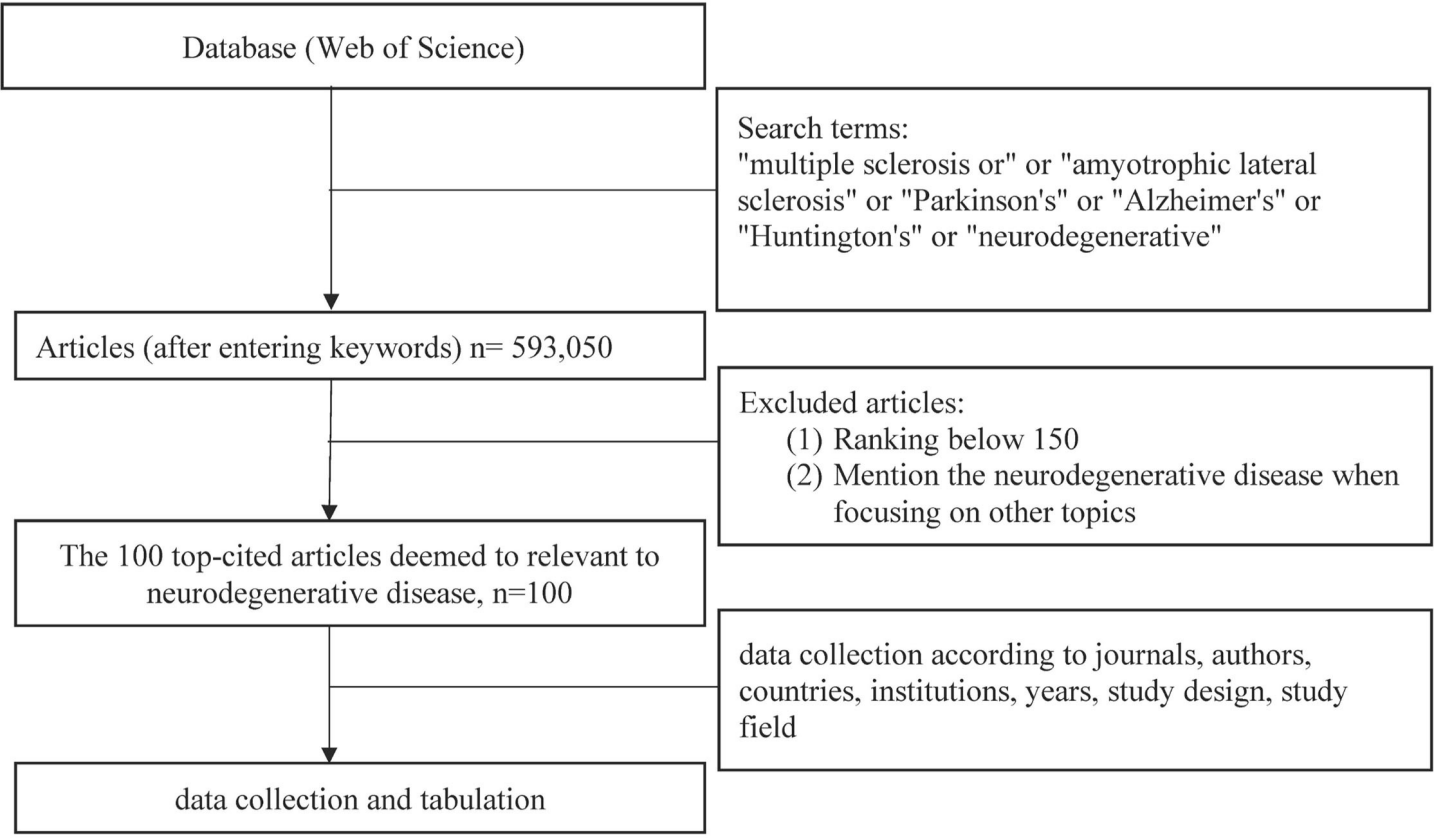
Flowchart of the methodology used in this study.

The articles were searched and gathered on July 22, 2020. A total of 593,050 articles were identified from 1950 to 2021 (Figure 1). There is an upper trend showed in the number of publications in the field of neurodegenerative diseases. In the last decade (2000–2009), 157,975 articles have been published, and the number doubled (318,864 articles) from 2010 to 2019.

### The Basic Characteristics of 100 Top-Cited Articles

The 100 top-cited articles were published from 1963 to 2013 and are listed in Table 1. Article citations range from 2,525 to 22,887 in total and 62 to 636 per year. The topic of the top 1 cited article is Alzheimer's diagnosis in 1984 (McKhann et al., 1984), which received 22,887 citations in total and 636 citations per year. The citations in 2019 and 2020 have been independently identified to show the present citation activity of highly cited articles, respectively, ranging from 43 to 1,424 and 24 to 799.

**Table 1:** Country, institute, and foundation with multiple articles in the most-cited list.

**Article characteristics**			**No. of articles**	**Citation/article**
Country		United States	68	4,333
		United Kingdom	9	3,794
		Japan	4	3,278
		Germany	3	5,130
		Canada	3	5,000
		France	3	3,283
Institute		Harvard University	8	4,147
		National Institutes of Health	8	4,947
		University of California	5	3,429
		Johns Hopkins University	4	3,827
		Washington University	4	3,460
Foundation	Governmental organization	NIH	49	3,856
		United States Department of Health & Human Services	4	4,142
		Medical Research Council UK (MRC)	3	3,473
	Companies	Bayer	8	4,157
		Pfizer	4	4,304
		Merck	4	5,119
		Eli Lilly	3	4,303
		Novartis	3	4,592
	Non-governmental organization	National Multiple Sclerosis Society	5	4,432
		Wellcome Trust	5	4,056
		Howard Hughes Medical Institute	3	4,898

### Publication Years of 100 Top-Cited Articles

**Figure 2:**
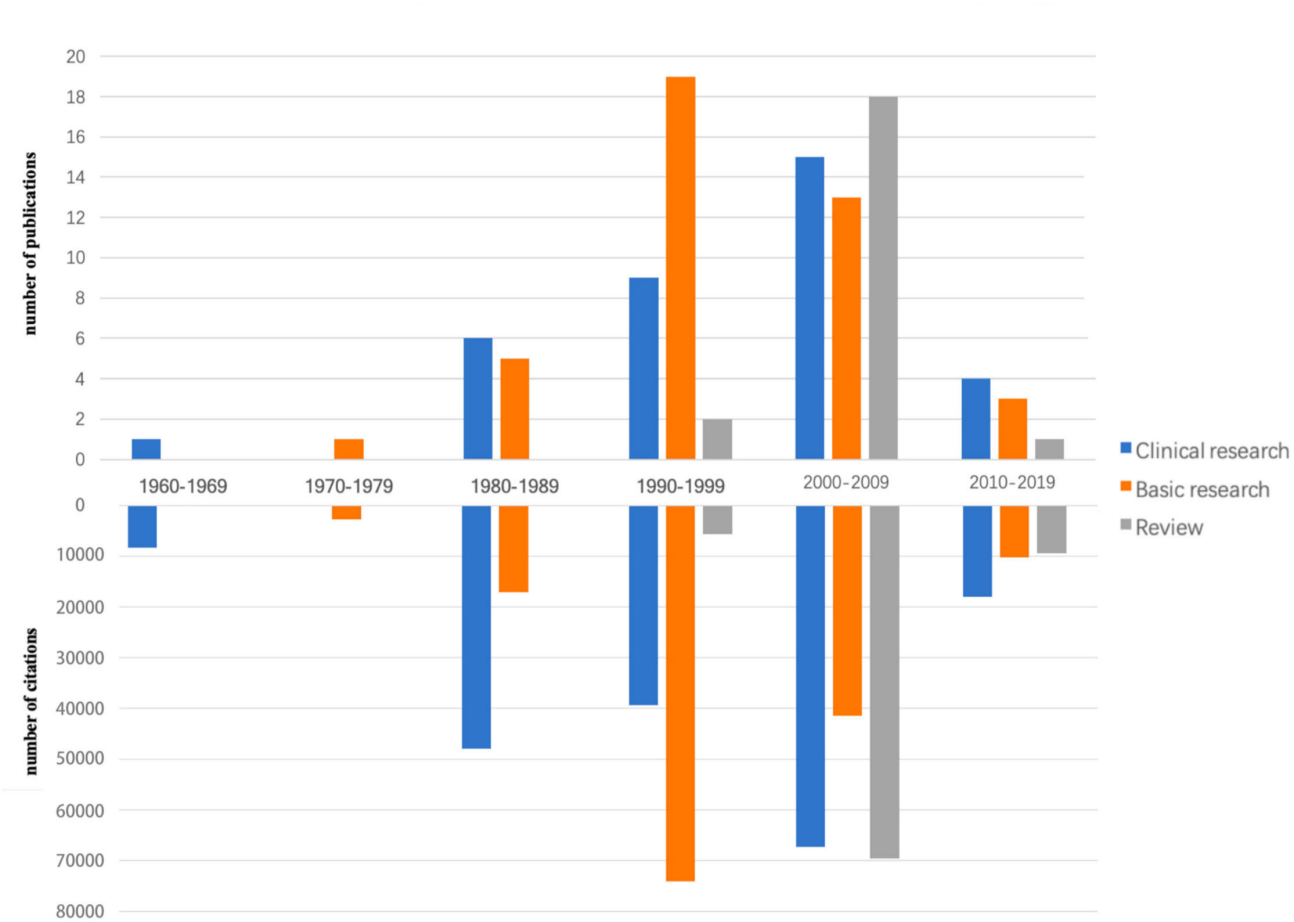
The total number of publications and citations according to a decade in 100 top-cited articles in neurodegenerative diseases.

Notably, 45% of the top-cited articles were published during 2000–2009, followed by 30 articles from 1990 to 1999 (Figure 2). After being divided into clinical (which involves clinical trials and other research protocols, and strictly controlled human studies of new therapies), basic research (which were performed in the laboratories using beakers and test tubes, not people. To help people better understand what causes a disease, to analyze how current treatments work, and to develop new potential therapies), and review, it showed that basic research reached a peak at 19 articles during 1990–1999 while clinical research peaked at 15 during 2000–2009. Before 1996, no relevant review is available while there is a great increase during 2000–2009 (i.e., 18 review articles in total). Most of the citations revealed the same trend as the number of publications, but the citation number in the 1980s is larger than that in the 1990's.

### Foundation, Country, and Institution of 100 Top-Cited Articles

Considering the origination of these 100 top-cited articles, the respondent authors of the majority of the publications were from the United States (*n* = 68) while nine publications came from the United Kingdom. Japan contributed four articles. Germany, Canada, and France published three articles each (Table 1).

Forty-seven articles have been funded by the National Institutes of Health (NIH), which belongs to the United States. Followed by Bayer, contributed 6 top-cited articles. National Multiple Sclerosis Society sponsored five articles while the MS topic only takes up 10% of the 100 top-cited articles. The top seven institutions in terms of the maximum number of articles were situated in the United States. Both Harvard University and NIH were associated with the same numbers of articles (eight of the 100 articles), followed by five articles from the University of California. Moreover, in the top 10 institutes with multiple articles, six of which are universities.

### Journal and Author of 100 Top-Cited Articles

Table 2 shows the top journals contributing maximum articles in the 100 top-cited articles in the field of neurodegenerative diseases. We categorized journals with multiple articles into general journals (*n* = 7) and specialty journals (*n* = 9). As our research focused on the field of neurodegenerative disease, we defined “Specify Journal” in this study as those journals that have been classified into “Neurosciences and Neurology” in Web of Science. Other journals that have been classified into “Science and Technology—Other Topics” have been regarded as general journals in our research. Journal SCIENCE [2020, Impact Factor (IF) = 47.728] contributed 17 articles, and the average citations of 17 articles are 4,058. While NATURE (2020, IF = 49.962) had 12 articles, followed by NEUROLOGY with nine articles. The average citations of the articles published in NEUROLOGY rank first at 6,636. In total, only 15 journals have more than two articles, but the average of citations has no huge difference, ranging from 2,690 from NEW ENGLAND JOURNAL OF MEDICINE to 6,836 from NEUROLOGY.

**Table 2:** Journals with multiple articles in the most-cited list.

**Category**	**Journals**	**No. of articles (%)**	**Citation/article**
General	SCIENCE	17	4,058
journals	NATURE	12	3,741
	CELL	5	4,271
	PROCEEDINGS OF THE NATIONAL ACADEMY OF SCIENCES OF THE UNITED STATE OF AMERICA	3	3,644
	LANCET	3	2,961
	NEW ENGLAND JOURNAL OF MEDICINE	2	2,691
Specialty	NEUROLOGY	9	6,836
journals	ANNALS OF NEUROLOGY	6	4,402
	PHYSIOLOGICAL REVIEWS	4	4,308
	ALZHEIMER'S AND DEMENTIA	3	4,304
	ARCHIVES OF NEUROLOGY	3	4,066
	NEURON	3	3,348
	NEUROBIOLOGY OF AGING	2	4,148
	JOURNAL OF INTERNAL MEDICINE	2	3,440
	CLINICAL NEUROPHYSIOLOGY	2	3,404

**Figure 3:**
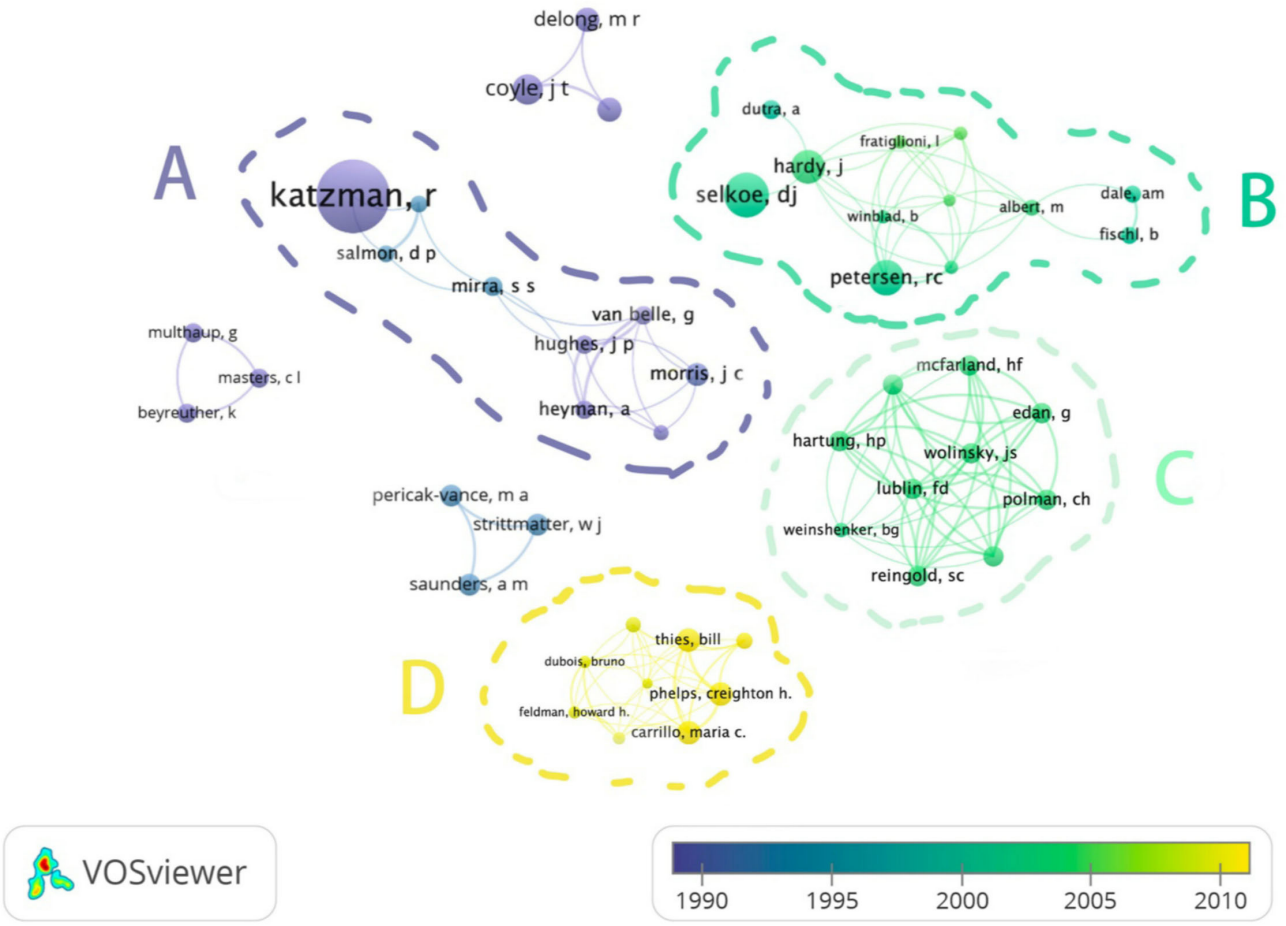
The connections between authors in 100 top-cited articles.

Figure 3 showed the connections and publication orders between authors. The 58 authors, who contributed to more than one article by VOSviewer, have been identified. To make a clear connection between authors, we hid the names of those authors, which have no connection or one link strength with others (*n* = 9). In cluster A, those authors published articles before 1995 and mainly focused on improving the clinical diagnosis. Cluster B has the largest connection, which included 12 authors. They came up with the amyloid hypothesis of AD around 2000. Cluster C included the author who mainly focused on making the criteria for the MD diagnosis. The latest articles in 100 top-cited articles were mainly published by the authors in cluster D. They contributed to defining the stages of AD, and some of them revealed the stage of the disease called “mild cognitive impairment.” To conclude, the clusters of authors reveal the evolution of research focus in neurodegenerative diseases.

### Study Topic and Study Type of 100 Top-Cited Articles

**Figure 4:**
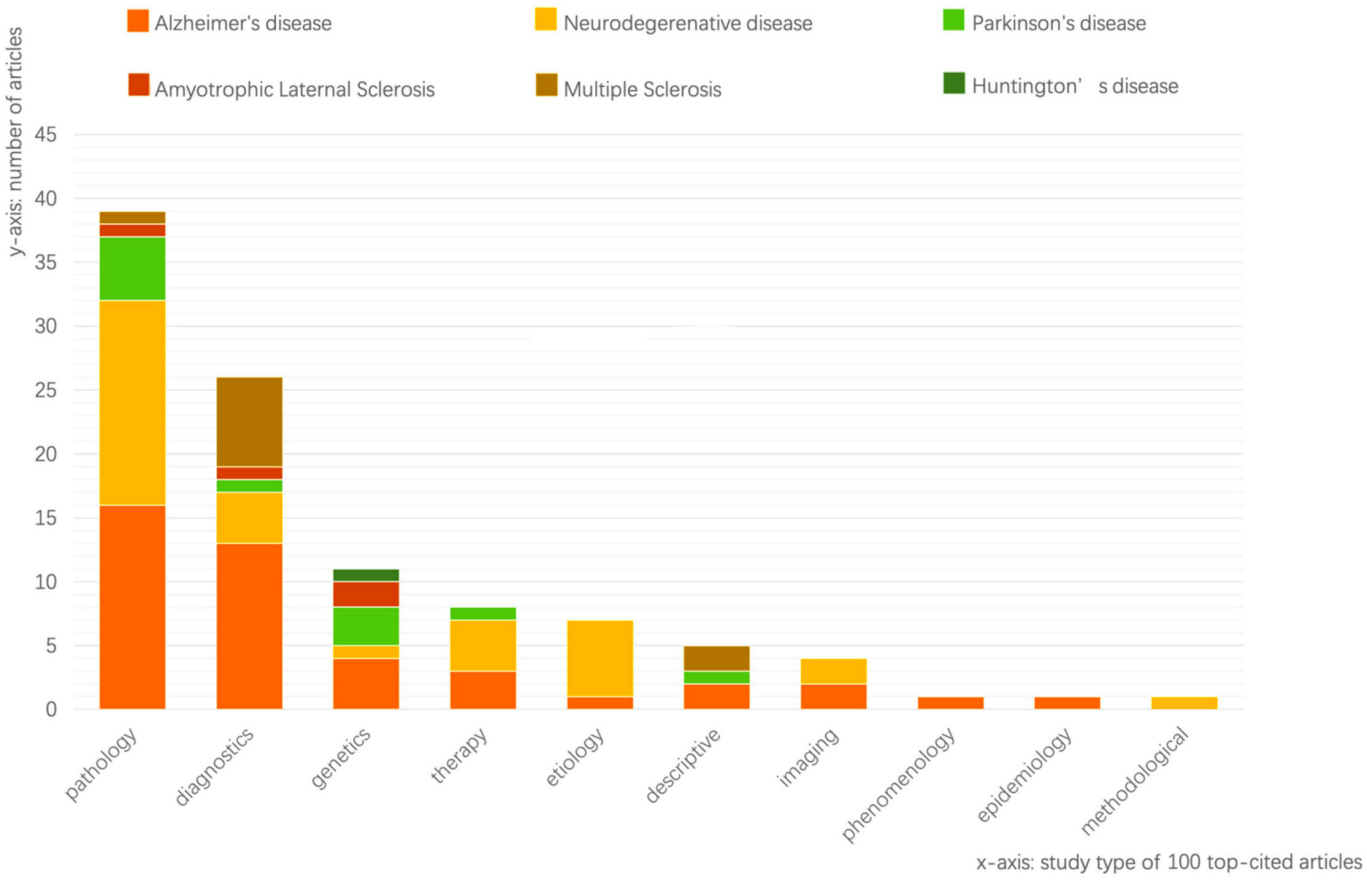
Statistics of the study topic and type of the 100 top-cited articles.

Among the 100 research articles, the articles with the topic of AD take the biggest part (*n* = 43), followed by 30 articles related to neurodegenerative diseases in general. Another two topics, PD and MS, respectively, contributed 11 and 10 articles. Only one article focuses on Huntington's disease (HD). However, there is an article about dementia with Lewy bodies shown in the 100 top-cited articles while this disease is not used as the search term. We classified the articles by our researchers according to the topic and the contents. We referred to some published articles to decide our study types (Nichols et al., 2004; Park et al., 2020). Study type in these 100 articles can be categorized into pathology (*n* = 39), etiology (*n* = 7), genetics (*n* = 11), imaging (*n* = 4), diagnosis (*n* = 23), and therapy (*n* = 8) (Figure 4); the type “descriptive” encompasses those articles, which in general describes a disease (e.g., Parkinsonism: onset, progression, and mortality; Mm and Yahr, 1967).

### Evidence Level of Clinical Articles (*n* = 22)

As most of the highly cited articles were published at the early stage of the research in the field of neurodegenerative diseases, basic researches take a bigger role in these 100 articles (*n* = 45), in which 22 clinical articles have been identified and categorized. Only one article belongs to evidence level 2 in the field of AD (published in 2004). While level 4 (case series with or without intervention; cross-sectional study) included six articles in total, there are 15 articles in evidence level 5. All the articles were about the diagnosis criteria from the opinions of respected authorities. The research focus is displayed in Table 3.

**Table 3:** Evidence levels of clinical articles in 100 top-cited articles in neurodegenerative diseases.

**Level**	**Rating criteria**	**No**.	**Corresponding article**	**Participants**	**Measurements**
1	Properly powered and conducted randomized clinical trial; systematic review with meta-analysis	0	-	-	-
2	Well-designed controlled trial without randomization; prospective comparative cohort trial	1	76	16 AD patients ranging in age from 51 to 81 years old	Whole-blood samples after injection of 18F-fluorodeoxyglucose(18-FDG)& PIB
3	Case–control studies; retrospective cohort study	0			
4	Case series with or without intervention; a cross-sectional study	6	4	802 patients exhibited some or all of the accepted cardinal signs	263 patients during the 2-year period
			79	Group1-19 men and 8 women with Alzheimer's disease and 26 normal subject group2-10 Alzheimer's subjects and 10 normal subjects	Two raters evaluated every subject to obtain inter-rater reliability of the diagnostic tool. A third interview was conducted 12 months after the initial session.
			6	94 patients meeting with mild cognitive impairment, 93 patients with mild Alzheimer's disease, 90 healthy elderly controls	The MoCA and MMSE of patients
			66	25 patients with chronic progressive MS with Expanded Disability Status Scale scores ranging from 3.0 to 6.5 29 patients with systemic lupus erythematosus 20 normal healthy adults.	28-item fatigue questionnaire
			13	76 consecutively evaluated subjects with mild cognitive impairment 234 healthy control subjects 106 patients with mild AD	Mini-Mental State Examination, Wechsler Adult Intelligence Scale–Revised, Wechsler Memory Scale–Revised, Dementia Rating Scale, Free and Cued Selective Reminding Test, and Auditory Verbal Learning Test were administered to all participants.
			7	100 consecutive cases clinically diagnosed invasive pneumococcal disease	Brains collected between June 1987 and August 1990
5	Opinion of respected authorities; case reports	15	8, 15, 19, 22, 34, 35, 40, 42, 59, 65, 69, 80, 87, 92, 95	-	-

## Discussion

Bibliometric analysis can provide enormous information with journals, institutions, authors, and countries, which is available for identifying landmark articles and high-impact journals (Shi et al., 2021). It not only provided a historical prospect in the field of neurodegenerative diseases but also revealed the trend of researches.

A few of the top-cited studies in the field of the neurodegenerative disease have been reported. In 2009, a bibliometric analysis related to AD has been reported by Sorensen. In 2018, Xue et al. reported the first time “The 100 most-cited articles in Parkinson's disease.” However, no top-cited bibliometric analysis has been conducted on the whole field of the neurodegenerative disease, which encompasses a broad array of conditions that include AD, PD, HD, MS, and ALS, with distinct clinical and pathological features. The articles of several citation analyses have been conducted on a specific disease [Aaron A conducted a citation analysis in the field of AD (Shen et al., 2019)]. However, neurodegenerative diseases share some common features, such as they are all related to progressive functional loss and neuron death within the central nervous system, also some intracellular protein degradation pathways (Laplante and Sabatini, 2012), such as autophagy. In order not to miss the researches on the common features of neurodegenerative diseases, our research encompasses all the neurodegenerative diseases.

The older an article is, the more likely it is cited (Jiang et al., 2016). Thus, articles published after 2010, while ranking top 100, might be the recent research hot spots. In our study, four of 10 articles, which were published after 2010, focused on improving the assessment tools of neurodegenerative diseases. Meanwhile, there is a turning point in the trend of citations while the number of publications increased decade by decade (Figure 2). It might mean the large outbreak happened in the 1980's and 2000's, which caused a huge increase in the number of citations.

In this study, the articles on neurodegenerative diseases from 1963 were bibliometrically analyzed. We found that all the articles are still been actively cited nowadays, especially in the diagnostic type. Similarly, the Montreal Cognitive Assessment (MoCA) got 799 citations during 2020, and the NINCDS-ADRDA (Nasreddine et al., 2005; Shen et al., 2019) (clinical diagnosis of AD) got 318 citations. The study type “diagnostic” takes up 23% of the 100 top-cited articles, but most of them are the opinions of respected authorities, and only one research conducted a clinical trial (Sorensen, 2009). Meanwhile, according to the result in VOSviewer (Figure 3), it also showed that the topic AD among neurodegenerative diseases attracted most of the researchers and has been studied internationally and collaboratively. Apart from the amyloid hypothesis, the other two major clusters were both focused on the diagnostic of AD. It might indicate that a lot of researches focused on the improvement of the diagnostic assessment tools in the field of neurodegenerative diseases. Moreover, the therapy articles are relatively less, and none of them conducted clinical trials. Thus, more effort should be paid to the studies on the therapy of neurodegenerative diseases other than AD.

We found that the United States confirmed the tremendous impact on the field of neurodegenerative diseases. The top five institutes with multiple articles cited are all located in the United States; meanwhile, the United States governmental organization NIH financially supported 49 types of studies. There is no doubt that the United States is a country with a research atmosphere, but it should also be concerned that most journals are from the United States, and the American reviewers possibly prefer US articles (Klunk et al., 2004). However, recently, other countries, such as Japan and China, have contributed significantly to global Alzheimer's research. Still, strengthening international cooperation could improve the quality and number of publications (Link, 1998).

The result of the journal analysis showed that the top-cited articles were published both in specialty journals (such as ALZHEIMER'S and DEMENTIA and NEUROBIOLOGY OF AGING) and in general journals (such as LANCET and NATURE). In the field of neurodegenerative diseases, specialty journals have attracted more attention to scientists in the past several decades (Dong et al., 2019). However, the journal that published most articles in the top-cited list is SCIENCE (*n* = 17), which is a general journal. This indicates that researchers should be encouraged to submit their articles to general journals that might attract the attention of more researchers from different fields.

A general limitation of our analysis is that bibliometric analysis is not an “exact science” and relies on interpretation and reiteration to achieve a “best fit” data set that will adequately describe the research area while excluding the articles of marginal relevance (Lasjaunias, 1999). We chose Web of Science because it is informally considered as the most accurate bibliographic source in the world, spans over 100 years, and includes several dozens of millions of publication records (Šubelj et al., 2014; Breugelmans et al., 2015). Another limitation is that the 100 top-cited articles are low-level evidence research because most of the highly cited articles were published at the beginning of the field, and some of them were raising important hypothesizes or diagnostic standards, which lack high-quality clinical evidence. In the future, instead of measuring the validity or the scientific quality of top-cited publications, the impact and the trend of the research might be focused more, and new analysis strategies, such as calculating the number of citations in the recent years and the number of citations per year, which showed the activeness of the articles, should be added to the basic analysis of the bibliometric studies.

## Conclusions

In conclusion, this study provided information on the top-cited articles in neurodegenerative diseases by using specific keywords for reference search in Web of Science. By systematically analyzed the quantity and quality of the articles in neurodegenerative diseases, it reveals that, first, the pathology and diagnostic researches took a major role in 100 top-cited articles while the therapy articles are relatively less. Second, the United States confirmed the tremendous impact on the field of neurodegenerative diseases. Third, researchers also submitted their researches to general journals, not just focused on specialty journals.

## Data Availability Statement

The original contributions presented in the study are included in the article/Supplementary Material, further inquiries can be directed to the corresponding author/s.

## Author Contributions

JS and ZL did all the revisions, wrote the latest versions, and screened the articles. XK, MY, and ZL extracted the data from each of the articles. MY, WW, and ZL performed the analysis and drafted the manuscript. JS supervised the project. All authors contributed to the conception and design of the study, commented on the previous versions of the manuscript, and read and approved the final manuscript.

## Funding

This study was financially supported by the Science and Technology Innovation Program of Hunan Province (2020RC2015), the China Postdoctoral Science Foundation (2021M693564), and the Natural Science Foundation of Hunan (2020JJ5865).

## Conflict of Interest

The authors declare that the research was conducted in the absence of any commercial or financial relationships that could be construed as a potential conflict of interest.

## Publisher's Note

All claims expressed in this article are solely those of the authors and do not necessarily represent those of their affiliated organizations, or those of the publisher, the editors and the reviewers. Any product that may be evaluated in this article, or claim that may be made by its manufacturer, is not guaranteed or endorsed by the publisher.
